# Education and training in adult metabolic medicine: Results of an international survey

**DOI:** 10.1002/jmd2.12044

**Published:** 2019-06-21

**Authors:** Annalisa Sechi, Elisa Fabbro, Mirjam Langeveld, Annarita Tullio, Robin Lachmann, Fanny Mochel, the SSIEM Adult Physicians Metabolic Group

**Affiliations:** ^1^ Regional Coordinator Centre for Rare Diseases Academic Hospital of Udine Udine Italy; ^2^ Department of Medicine University of Udine Udine Italy; ^3^ Academic Medical Centre University of Amsterdam Amsterdam The Netherlands; ^4^ Institute of Hygiene and Clinical Epidemiology Academic Hospital of Udine Udine Italy; ^5^ Charles Dent Metabolic Unit, NHNN London UK; ^6^ Reference Center for Neurometabolic Diseases La Pitié‐Salpêtriere University Hospital Paris France

**Keywords:** adult metabolic medicine, education and training, inherited metabolic diseases, survey

## Abstract

Adult metabolic medicine (AMM) is an expanding medical subspecialty, due to the increasing number of adult patients with inherited metabolic diseases (IMD). However, a formal training and postgraduate education in this field is not available in the majority of countries. Existing adult metabolic specialists (AMS) come from many different backgrounds. The aim of this survey was to assess the state of play as regards education and training in AMM worldwide. Members of the Society for the Study of Inborn Error of Metabolism adult metabolic group (n = 89) were asked to take part in this survey. Forty‐two AMS (47.2%) from 18 different countries completed the questionnaire. The most common specialties were internal medicine (38.1%), endocrinology (26.2%), genetics (21.4%), and neurology (21.4%). Ninety‐five percent of respondents considered that practical clinical experience had contributed importantly for their professional development, while only 27% felt the same for formal academic education. The current state of available education and training was judged as generally poor or fair (73% of the respondents). The most suggested ways of improving education and training in AMM were: to facilitate international internships; to implement courses on adult‐IMD; and to create a formal academic education. The skills considered most important for AMS were: recognition of signs and symptoms of diseases, knowledge of the available treatments, and ability to perform a correct follow up. In conclusion, worldwide, current available education and training in AMM is considered inadequate. This survey emphasizes the need for development of new, formal training opportunities to improve knowledge, and competence in this rapidly expanding field.

## 1 INTRODUCTION

Adult metabolic medicine (AMM) is a relatively new medical specialty, focusing on diagnosing, managing, and researching inherited metabolic diseases (IMD) in adults. It includes the care of both patients diagnosed with an IMD during childhood who survive into adulthood, and patients who are diagnosed in adulthood. These patients represent an expanding population. Thanks to better diagnosis and pediatric care over the last 30 years, patients are living longer. Improved metabolic and genetic diagnostic tools have also led to the detection of milder forms of many conditions, which present in adulthood. Moreover, through the expanding newborn screenings affected parents of the babies are increasingly detected. All these patients need long‐term follow up.^1^, ^2^ A recent survey of the metabolic European reference network showed that 50% of patients with IMD are adults (Scientific report, Board annual meeting 2018, MetabERN). Adult metabolic patients have their own peculiarity, compared to pediatric ones, showing an high phenotype variability depending not only from genotype, but also from modulation in response to environment modifications and to ontogenic changes and aging.^3^

In the past, metabolic medicine has been considered a pediatric discipline, and no formal postgraduate training or education in AMM exists in the majority of countries. As a result, current adult metabolic specialists (AMS) come from varied backgrounds. In 2010, as part of the Society for the Study of Inborn Error of Metabolism (SSIEM), a group of physicians with a special interest in AMM founded the Adult Metabolic Physicians Working Group (http://www.ssiem.org/amp/welcome.asp). The group meets every year at the annual SSIEM meeting for a specific session on IMD in adults, and a mailing list of members has been set up. The aim of this study was to describe the current landscape of education and training in AMM worldwide, and explore what steps are needed to further strengthen and establish the specialty of AMM.

## 2 METHODS

A survey (see Supporting Information) consisting of 11 questions, divided into three sections—the participants qualifications and training in AMM, the availability and quality of education and training in AMM, and their opinion of the competencies required in an AMS—, was developed by the authors. AMS that are members of the SSIEM adult metabolic group (89 members) were contacted by email and asked to fill out the survey. Data were collected between July and December 2017.

Descriptive statistics were performed on categorical and numerical variables. Frequency distributions were used for categorical variables. For numerical variables, mean, median, interquartile range, SD, 25° and 75° percentile, minimum and maximum values were considered. Kolmogorov‐Smirnov test was performed for checking data distribution's normality on numerical variables. When variables were not normally distributed, nonparametric tests were performed and the median ± interquartile range were considered, for normally distributed variables mean ± SD were considered and parametric tests were performed. For question 1.2, the replies were first considered as dichotomous data (Yes or No); then, for the replies that have been answered as Yes, the replies were considered as categorical data (from 1‐not important to 4‐very important). For question 1.4, the percentage of adult patients (>16 years) affected by IMD was compared to the total amount of patients followed by participants. Fisher's exact test was performed to search for differences in scores from question 2.1 based on replies to question 1.2, and on replies to question 1.3 considered as dichotomous data (ie, working experience in AMM ≤ or >10 years). All statistical analyses were performed using SAS software, version 9.4 (SAS institute, Inc, Cary, North Carolina) and R 3.4.2. The significance level was set at .05.

## 3 RESULTS

Forty‐two AMS (47.2%) from 18 different countries and 5 different continents (34 from Europe, 3 from North America, 2 from South America, 1 each from Africa, Asia, and Oceania) completed the questionnaire.

**Figure 1:**
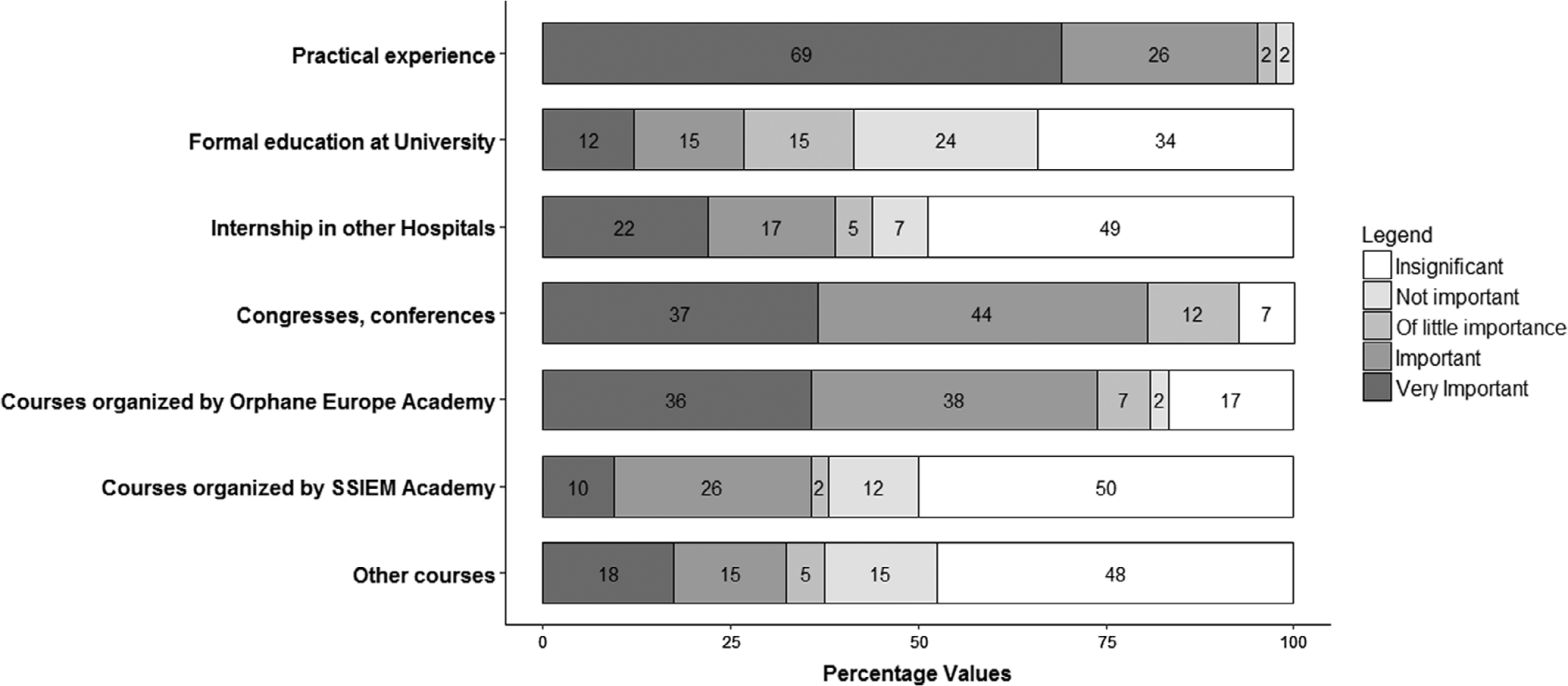
Participants' education and training in adult metabolic medicine

Participants were mostly specialists in internal medicine (38.1%), endocrinology (26.2%), genetics (21.4%), and neurology (21.4%). Fifteen out of 42 participants had more than one speciality. Four participants, working in the United Kingdom and Portugal, were officially recognized as metabolic specialists by their healthcare systems. No similar qualification was recognized in the other countries. Regarding working experience in AMM, 47.6% of participants were working in this field for more than 10 years, 35.7% for 5 to 10 years, 16.7% for less than 5 years. The average IMD patients load, as proportion of the total patient load of the participants was 54.4% ± 37.0%. Ninety‐five percent of AMS considered that day to day practical experience in their own unit had been important or very important for their professional development, while formal academic education or training programs had contributed importantly for only 27% of the respondents and not importantly or insignificantly for 58%. Congresses and conferences contributed importantly or very importantly for 81%. Other answers are reported in Figure 1. Fourteen out of the 42 participants (33.3%) had been able to do an IMD internship in a foreign country, lasting from 1 month to 2 years.

The quality of available education and training in AMM was judged as generally poor or fair (73% of respondents). However, there were discrepancies among countries. Physicians from Portugal, France, Ireland, the United Kingdom, and the United States had a more positive opinion of the training available in their countries (data not shown). These training programs seem to have improved over time as younger AMS, with fewer years of experience in the field, tended to have more positive perceptions. In general, participants with more than 10 years of experience in AMM, compared to those with less than (or equal to) 10 years of experience had a less positive opinion of the available training (*P* = .038).

The available country‐specific training programs in AMM, as reported by participants, are summarized in Table 1.

**Table 1:** Available specific training programs in AMM, reported by participants

Country	City	Title	Target population	Level (National/regional/local)	Language	Duration	Website
Australia	Sydney	Full time metabolic fellowship across Children's Hospital WestMead and WestMead Hospital (adult)	Senior registrars in pediatric and adult metabolic medicine, or specialists (not specific for adult IEM)	Regional (NSW state‐wide service)	English	1 year, full time	Not applicable
Canada	Vancouver	National biochemical genetics training program	Residents in genetics, pediatrics, internal medicine, and biochemistry	National	English	1 month, full time adult exposure; total duration 24 months	https://www.ccmg‐ccgm.org/training/training‐clinical.html
France	Paris	Inter‐University Diploma in inherited metabolic diseases	Residents in pediatrics, genetics, neurology, internal medicine, endocrinology, hepatology, and biochemistry	National	French	104 teaching hours, 6 weeks practical training	http://www.scfc.parisdescartes.fr/index.php/descartes/formations/medecine/pediatrie‐medecine‐de‐l‐adolescent/diu‐maladies‐hereditaires‐du‐metabolisme/(language)/fre‐FR
Ireland	Dublin	Special interest in metabolic diseases	Residents in genetics or fellows	National	English	2 years	https://www.mater.ie/services/adult‐metabolic‐service/
Netherlands	Rotterdam/Amsterdam	Training in adult metabolic medicine for internal medicine residents	Residents in internal medicine	National	Dutch	4 months full time	Not applicable
Netherlands	Rotterdam/Amsterdam	Training in adult metabolic medicine for endocrinology fellows	Endocrinology fellows (senior residents)	Regional	Dutch	6 months part time (1 day a week)	Not applicable
Portugal	Porto	Ciclo de Estudos Especiais em Doenças Hereditárias do Metabolismo	Specialists pediatricians or internists	National	Portuguese	2 years, full time	https://dre.pt/
United Kingdom	London, Birmingham, Manchester, Cambridge, Cardiff, Glasgow	Metabolic medicine training program	Subspecialty for residents in chemical pathology and internal medicine	Regional	English	5 years, full time	www.jrcptb.org.uk

Abbreviation: AMM, Adult metabolic medicine.

**Figure 2:**
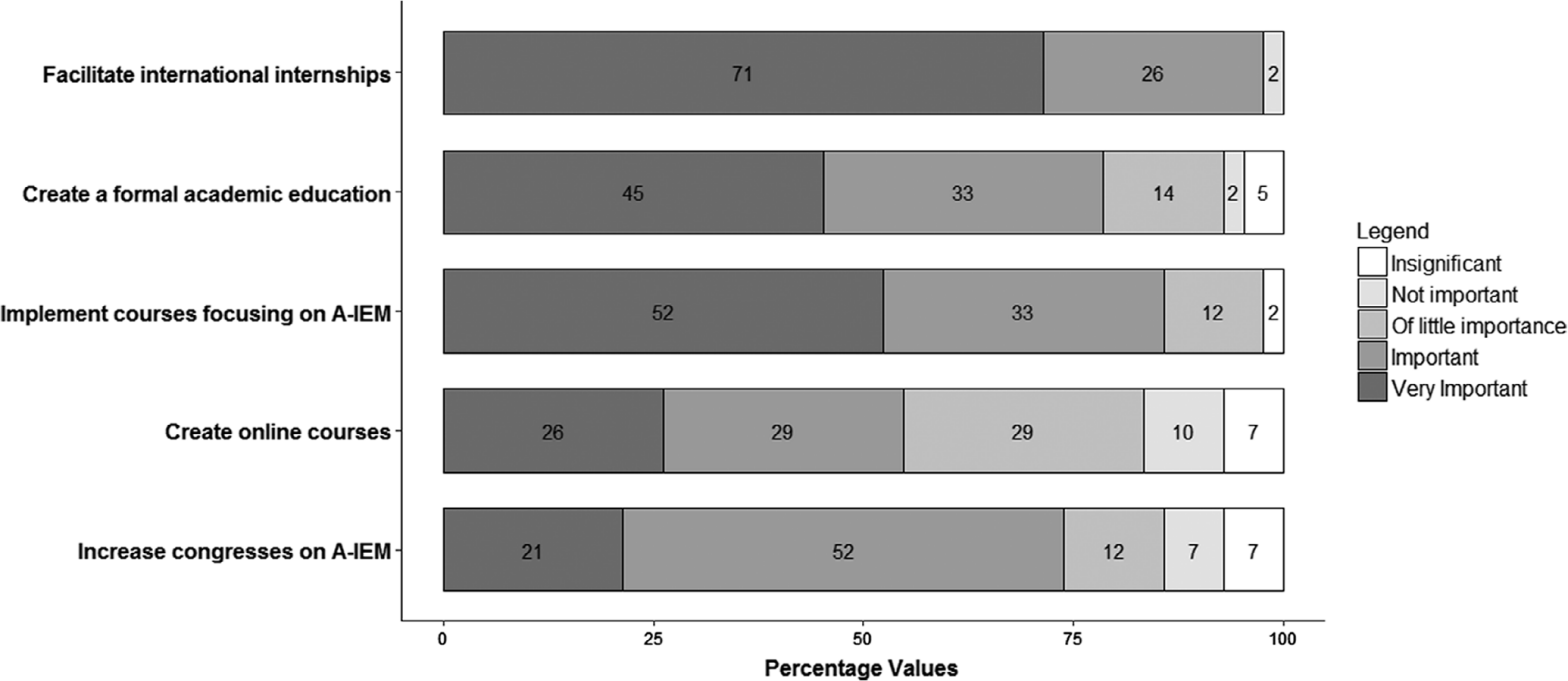
Suggested ways to improve education and training in adult metabolic medicine

When asked about possible ways to improve education and training in AMM, participants suggested: facilitating international internships in centers with experience in AMM (considered very important or important by 97%), implementing courses on IMD focusing on adults (85%) and establishing formal academic education in AMM (78%; Figure 2).

**Figure 3:**
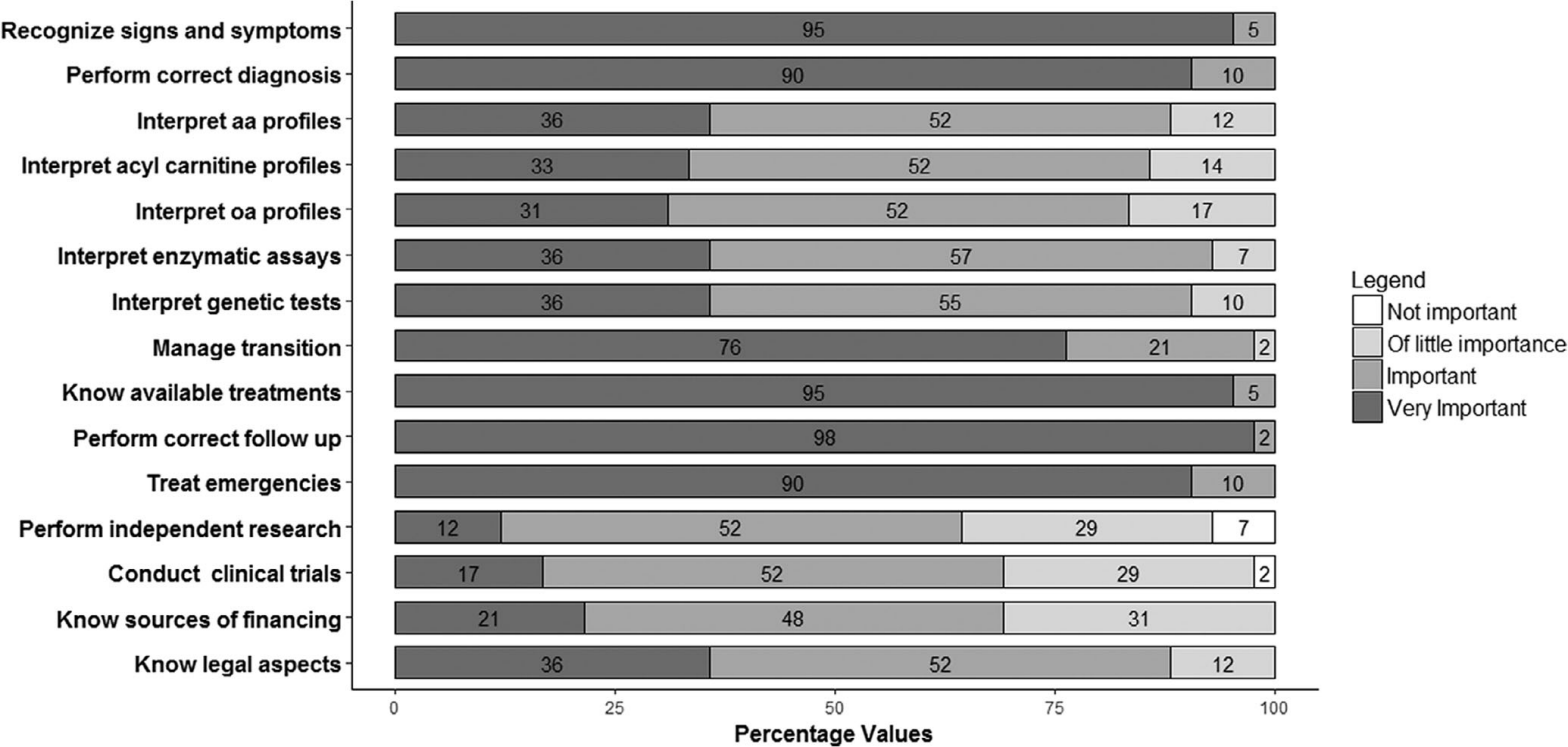
Competence of an adult metabolic specialist

The most important competencies for AMS were thought to be: recognition of signs and symptoms suggestive of an IEM (considered very important by 95%), knowledge of available treatments (95%), and ability to perform correct follow up (98%). Other answers are reported in Figure 3.

## 4 DISCUSSION

AMM is an expanding medical specialty. A previous survey by the SSIEM Adult Metabolic Physicians Working Group assessed the workload of the main international centers specializing in AMM and showed that the number of patients being referred to these services is increasing year on year. Adult metabolic patients in this study were affected by a wide spectrum of diseases, including in order of frequency amino acid, lysosomal, mithocondrial, peroxisomal, glycogen storage, fatty acid oxidation disorders, porphyrias and vitamin, and cofactor disorders. While many patients were being transitioned from pediatric metabolic services, more than 40% were only diagnosed in adulthood, demonstrating the need for adult physicians to be aware of these diseases.^2^

The physicians who participated in the current survey were also identified through the Adult Metabolic Physicians Working Group of the SSIEM, and are representative of leading centers that take care of adult patients. Among respondents, 83.3% had been working in AMM for more than 5 years. On average, these specialists were spending 54% of their time on adult patients with IEM. Despite this, only 4 out of the 42 responders were officially designated as specialists in AMM by their healthcare systems. Hence, in many countries, the role of AMS is not officially recognized, and no formal education or training in AMM exists. Unsurprisingly, as IMD in adulthood are often multisystem disorders,^4^ the specialties which the respondents came from varied, although internal medicine and endocrinology were most frequent. Probably because of the lack of formal training, the vast majority of respondents considered that their training in AMM had mostly been based on practical experience rather than academic education. Seventy‐three percent of the participants felt the current state of available education and training in AMM was insufficient.

These data highlight the fact that currently it is difficult to obtain the basic knowledge and skills required to manage adult patients with IMD unless and until one is in post as an AMS. The corollary of this is that it is difficult to find young physicians who have sufficient knowledge and skills to appoint to a new AMS post. This situation is not sustainable and has been recognized by patients' associations.^5^ Bodies such as the European Commission have acknowledged the need to develop and implement effective training programs for rare disease specialists.^6^, ^7^ Because of the lack of suitably trained AMS, many adult patients who were diagnosed with IMD as children remain under the care of pediatricians. This is not viable in the long term: pediatric settings are not suitable for adult patients who in any case develop very different needs as they age (autonomy, pregnancy, comorbidities associated with aging).^4^ It is critical that these patients can be transitioned to AMS. The timing of transition is not easy to define, especially for patients who may develop severe visceral or neuropsychiatric complications, which requires the identification of the right adult structures and professionals for care continuity.^8^ Some countries, such as Portugal, UK, France, and the Netherlands^9^ have already made important advances in this direction. The availability of educational opportunities dedicated to AMM is increasing, as testified by Table 1. This means that the new generation of specialists will have more opportunities to train than the previous one. This education is also perceived to be of improved quality.

The results of the second and third part of this survey should help those trying to develop training programs in AMM. Trainees need the opportunity to gain hands on clinical experience, while the more obvious biochemical skills, such as interpreting diagnostic tests, were felt to be less important as they were well‐performed by specialist metabolic laboratories. The actions that were deemed important (facilitating international internships, providing courses on adult‐IMD, and creating a formal syllabus for education) will require funding and support from healthcare systems and professional societies. A further survey aimed at identifying which AMM centers can accept trainees from other countries would help in organizing international internships. A panel of experts in AMM could be set up to discuss an educational curriculum and how to organize and accredit training in AMM. Existing models, as used in the United Kingdom, where formal training including AMM already exists (either as a subspecialty of internal medicine or chemical pathology), could be adopted more widely. However, other options should also be considered, including the creation of a completely new medical specialty or as a credential for doctors trained in other specialities. Experience from other medical specialties, who have faced the problem of how to harmonize and implement training in developing disciplines, suggest that establishing an e‐learning platform can be effective (for example, infection control training for healthcare workers,^10^).

Finally, as adult metabolic diseases are often multisystem disorders and can show various complications, the AMP should work in a multidisciplinary team, involving other specialists such as nephrologist, cardiologist, gastroenterologist, and other healthcare professionals such as specialized nurses, dieticians, physical therapist, and psychologists. All these professionals should have adequate training in AMM, thus courses and congresses on AMM open to all these categories should be implemented.

## 5 CONCLUSIONS

Worldwide, current available education and training in AMM is inadequate. There is a need for new, formal training programs to improve knowledge and competence in this rapidly expanding specialty.

## CONFLICT OF INTEREST

Annalisa Sechi, Elisa Fabbro, Mirjam Langeveld, Robin Lachmann, and Fanny Mochel declare that they have no conflict of interest.

## ETHICAL APPROVAL STATEMENT

All procedures followed were in accordance with the ethical standards of the responsible committee on human experimentation (institutional and national) and with the Helsinki Declaration of 1975, as revised in 2000. This article does not contain any studies with animal subjects performed by the any of the authors.

## PATIENT CONSENT STATEMENT

Informed consent was not obtained for each participant in this study.

## Supporting information

supplementary information
