# Supplementary material for: Education and training in adult metabolic medicine: Results of an international survey
Source: JIMD Rep. 2019 Jun 21;49(1):63–9. doi: 10.1002/jmd2.12044 (PMC6718119; doi:10.1002/jmd2.12044)
Supplement: Supplementary file 1 — supplementary information [file JMD2-49-63-s001.pdf]

Adult Metabolic Specialists Survey

Date of compilation:	
Name:	
Surname:	
Date of birth:	
Hospital/Institution:	
Department/Division:	
City:	
Country:	
e-mail address:	
Phone:	

SECTION 1: Personal curriculum

1. Specialization:

- ☐ internal medicine
- ☐ endocrinology
- ☐ neurology
- ☐ genetics
- ☐ pediatrics
- ☐ none
- ☐ others (specify) ___________________________

1.1 In which University/Hospital? ______________________

2. How did you get education and training in adult metabolic medicine?

Please, rate the importance for the positive answers:	Not important	Of little importance	Important	Very Important
☐ Practical experience in my Unit	①	②	③	④
☐ Formal education at University<br>*Please, specify name and place of the University*:<br>____	①	②	③	④
☐ Internship in other Hospitals<br>*Please, indicate place, year and duration:*<br>____	①	②	③	④
☐ Congresses, conferences and symposia	①	②	③	④
☐ Courses organized by Orphan Europe Academy (Recordati Rare Diseases Foundation)<br>*Please indicate the number of courses attended:*<br>____	①	②	③	④

☐ Courses organized by SSIEM academy<br>*Please indicate the number of courses attended:*<br>____________________________________	① ② ③ ④
☐ Courses organized by other professional bodies<br>*Please specify the title of the course, giving organization and duration in h*<br>____________________________________	① ② ③ ④
☐ Others<br>*Please, specify*<br>____________________________________	① ② ③ ④

3. How long have you been working in adult metabolic medicine?

☐ 1-5 years ☐ 6-10 years ☐ >10 years

4. What is the total amount of patients currently followed by yourself? **…………….**

4.1 How many of them are affected by inherited metabolic diseases? **…………….**

4.2 How many of those with inherited metabolic diseases are adults (>16 y)? **…………….**

5. How is your Unit organized?

5.1 Number of doctors: **….** 5.2.1 How many take care of adult metabolic patients? **….**

5.2 Number of nurses**:** **….** 5.2.1 How many with experience in metabolic drugs? **….**

5.3 Number of dieticians with experience in metabolic medicine: ….

5.4 Other professionals involved in adult metabolic medicine (specify professional figures and number)____

5.5 Have you got a metabolic laboratory in your Hospital? ☐ yes ☐ no

5.5.1 If yes, which test do the laboratory perform?

☐ aminoacid profile ☐ acylcarnitine profile ☐ organic acid profile ☐ enzyme assays

☐ genetic tests

6. In your Unit have you ever had doctors in traineeship in adult metabolic medicine?
   ☐ yes ☐ no

   6.1 If yes, how many of them in the last 5 years? ………………

   6.2 From where (which country) did they come? ___________________________________

SECTION 2: Existing education-training

1. How do you evaluate the state of education and training in adult metabolic medicine available for a beginner with a strong interest in this field:

Please, rate the importance for the positive answers:	Poor	Fair	Good	Very good
☐ In your Country	①	②	③	④
☐ In general	①	②	③	④

2. Are there specific training programmes in adult metabolic medicine in your country?

☐ Yes. *Please, specify the level:*
- ☐ International level
- ☐ National level
- ☐ Regional level
- ☐ Local level

☐ No
☐ Unknown

2.1 If yes to question 2, please describe them filling in the following scheme:									
	Title of training programme	Organizational Level (National/ Regional/Local)	Responsible organisation (contact details)	Language	Number of hours per training programme	Number of hours of theorical training	Number of hours of practical training	Does the course include the possibility of distance learning? Y or N	Website Link
1									
2									
3									
4									
5									

3. In your opinion, what would be the way to improve education and training in adult metabolic medicine?

Please, rate the importance for the positive answers:	Not important	Of little importance	Important	Very Important
☐ Facilitate international internships in Units with experience in adult metabolic medicine	①	②	③	④
☐ Create a formal academic education in adult metabolic medicine giving a specific title (i.e. a master degree)	①	②	③	④
☐ Implement existing courses on rare metabolic diseases focusing more the topic on adults	①	②	③	④
☐ Create online courses	①	②	③	④
☐ Increase the number and the quality of congresses on adult metabolic medicine	①	②	③	④
☐ Others (specify):	①	②	③	④

4. Does your employment system guarantee to health care workers any leaves for personal education?

☐ yes ☐ no ☐ unknown

If Yes, Please specify the amount of hours/year **………………**

SECTION 3: Competence of an adult metabolic specialist

In your opinion, what skills should have an adult metabolic physician?

Please, rate the importance for the positive answers:	Not important	Of little importance	Important	Very Important
☐ Recognize signs and symptoms suggestive for an adult metabolic disease	①	②	③	④
☐ Perform the correct diagnostic process	①	②	③	④
☐ Interpret the results of aminoacids profile	①	②	③	④
☐ Interpret the results of acil carnitine profile	①	②	③	④
☐ Interpret the results of acid organic profile	①	②	③	④
☐ Interpret the results of enzyme assays	①	②	③	④
☐ Interpret the results of genetic tests	①	②	③	④
☐ Manage the transition from pediatric care to adult care	①	②	③	④
☐ Know available treatments and their management	①	②	③	④
☐ Perform a correct follow up	①	②	③	④
☐ Treat emergencies	①	②	③	④
☐ Perform independent research	①	②	③	④

☐ Conduct clinical trials	① ② ③ ④
☐ Be aware of the main source of financing	① ② ③ ④
☐ Know the legal aspects	① ② ③ ④
☐ Others (specify):<br>____<br>____	① ② ③ ④
